# Neurological syndrome with CSF anti-Hu positivity during serplulimab therapy in extensive-stage small-cell lung cancer: a case report

**DOI:** 10.3389/fonc.2026.1974779

**Published:** 2026-10-26

**Authors:** Linjing Wang, Yanan Zhao, Bo Fan, Shang Sang, Xinying Li, Jia Yu, Yuansong Bai

**Affiliations:** 1 Department of Medical Oncology, China-Japan Union Hospital of Jilin University, Changchun, Jilin, China; 2 Second Ward, Department of Medical Oncology, Xinyang Central Hospital, Xinyang, China

**Keywords:** anti-Hu antibody, immune checkpoint inhibitor, intestinal pseudo-obstruction, neurological toxicity, paraneoplastic neurological syndrome, serplulimab, small-cell lung cancer

## Abstract

**Background:**

Immune checkpoint inhibitors (ICIs) have improved outcomes in extensive-stage small-cell lung cancer (ES-SCLC), but neurological immune-related adverse events (n-irAEs) can be severe and disabling. Small-cell lung cancer (SCLC) is also strongly associated with anti-Hu-associated paraneoplastic neurological syndromes (PNSs). Neurological syndromes emerging or worsening after ICI exposure may therefore represent post-ICI PNS, in which paraneoplastic autoimmunity and ICI-related neurotoxicity overlap clinically.

**Case presentation:**

A 52-year-old woman with stage IV ES-SCLC received four cycles of serplulimab plus etoposide and carboplatin, followed by five cycles of serplulimab maintenance. During maintenance therapy, she developed anorexia, weight loss, and alternating constipation and diarrhea, followed by progressive memory impairment, somnolence, cognitive dysfunction, and gait instability. Cerebrospinal fluid (CSF) examination showed mild mononuclear pleocytosis, and CSF anti-Hu antibody was detected by a cell-based assay at a titer of 1:100; no residual specimen was available for independent confirmatory testing. Brain magnetic resonance imaging (MRI) demonstrated newly developed bilateral paramedian thalamic abnormalities, whereas diffusion-weighted imaging and apparent diffusion coefficient mapping showed no evidence of acute cerebral infarction. The neurological presentation was classified as an intermediate-risk phenotype under the 2021 PNS-Care criteria. If the reported CSF anti-Hu result is accepted, the recalculated PNS-Care score would be 9, corresponding to the definite PNS category, although interpretation is limited by the absence of orthogonal antibody confirmation. Serplulimab was permanently discontinued, and corticosteroids plus intravenous immunoglobulin (IVIG), followed by individualized IVIG maintenance, resulted in substantial but incomplete neurological recovery. MoCA, MMSE, and mRS improved from 11/30, 12/30, and 3 to 24/30, 22/30, and 2, respectively, although residual cognitive impairment persisted. Separately, a complete response was first documented on July 20, 2021, and radiological CR remained confirmed through May 2023, with no subsequently documented clinical tumor progression before death in January 2025. In 2024, intestinal pseudo-obstruction required segmental colectomy, and histopathology demonstrated lymphocytic inflammation surrounding the myenteric plexus.

**Conclusion:**

This case illustrates the diagnostic overlap between paraneoplastic neurological autoimmunity and ICI-related neurological toxicity in a patient with CSF anti-Hu positivity. The later gastrointestinal episode raised the possibility of enteric nervous system involvement, but its etiology remained uncertain.

## 1 Introduction

Immune checkpoint inhibitors (ICIs) combined with platinum-etoposide chemotherapy have become a standard first-line treatment for extensive-stage small-cell lung cancer (ES-SCLC) and have improved survival in a disease historically associated with poor long-term outcomes (1). However, immune activation may disrupt immune tolerance and result in immune-related adverse events (irAEs). Neurological immune-related adverse events (n-irAEs) are uncommon but clinically important because they may progress rapidly and cause persistent neurological disability or death (2). The overall clinical course is summarized in **Figure 1**.

**Figure 1:**
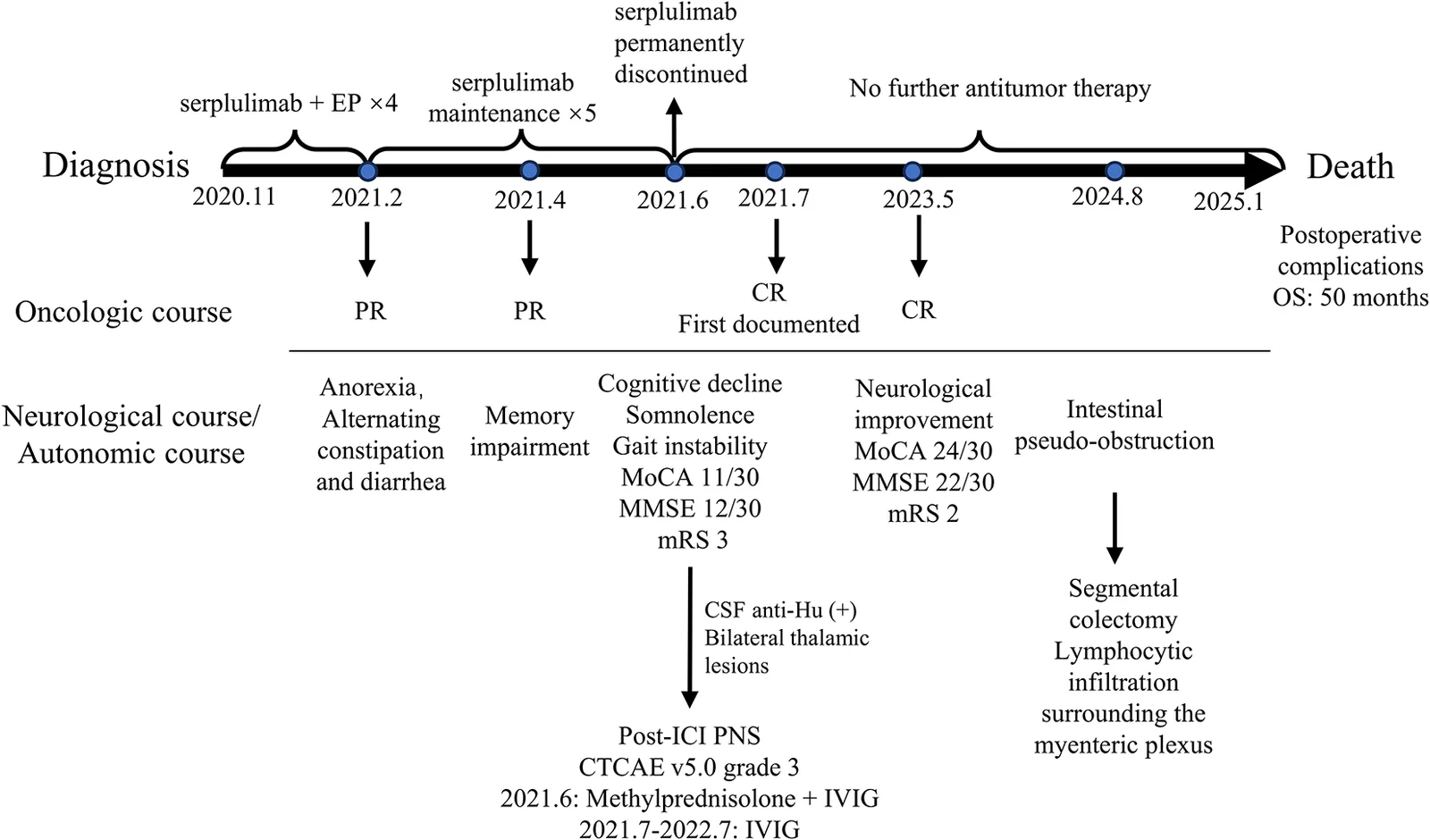
Clinical timeline of the disease course. Timeline showing the diagnosis of extensive-stage small-cell lung cancer, four cycles of serplulimab plus etoposide and carboplatin, five cycles of serplulimab maintenance, gastrointestinal symptoms, onset and progression of neurological manifestations, cerebrospinal fluid and magnetic resonance imaging findings, anti-Hu antibody detection, corticosteroid and intravenous immunoglobulin treatment, permanent discontinuation of serplulimab, tumor response, neurological recovery, intestinal pseudo-obstruction, and final outcome. Complete response was first documented on July 20, 2021. Radiological complete response was confirmed through May 2023, with no subsequent clinical evidence of progression before death in January 2025.

Small-cell lung cancer (SCLC) is also one of the malignancies most strongly associated with paraneoplastic neurological syndromes (PNSs), particularly anti-Hu-associated neurological disease. Hu antigens are intracellular neuronal RNA-binding proteins that are physiologically expressed in nervous tissue but may also be aberrantly expressed by SCLC cells, thereby providing a shared antigenic substrate for antitumor and neuronal autoimmune responses (3, 10). Anti-Hu-associated PNS can involve the central, peripheral, autonomic, and enteric nervous systems (10).

Blockade of the programmed cell death protein 1 (PD-1)/programmed death-ligand 1 (PD-L1) pathway may release inhibitory constraints on autoreactive T cells and amplify previously subclinical paraneoplastic autoimmunity. PNS developing *de novo* or worsening after ICI exposure has been described as post-ICI PNS, a clinically useful term that emphasizes the temporal association between checkpoint inhibition and paraneoplastic autoimmunity without implying that ICIs are necessarily the sole cause of the neurological syndrome (3–5).

Here, we report a patient with ES-SCLC who developed a rapidly progressive neurological syndrome with CSF anti-Hu positivity during serplulimab treatment, preceded by gastrointestinal symptoms and followed several years later by intestinal pseudo-obstruction with lymphocytic inflammation surrounding the myenteric plexus.

## 2 Case description

### 2.1 Patient information and oncological treatment

A 52-year-old woman with no relevant medical or family history was diagnosed on November 16, 2020, with right-sided SCLC. Initial staging had been performed by PET/CT at an outside hospital; however, the original PET/CT images and report were unavailable for retrospective review. Baseline cervical MRI obtained at our institution demonstrated abnormal right level II and level IV cervical lymph nodes. Follow-up cervical MRI on May 12, 2023, showed no pathological cervical lymphadenopathy (**Figure 2**). The disease was clinically staged by the treating team as T1bN3M1b, stage IV ES-SCLC.

**Figure 2:**
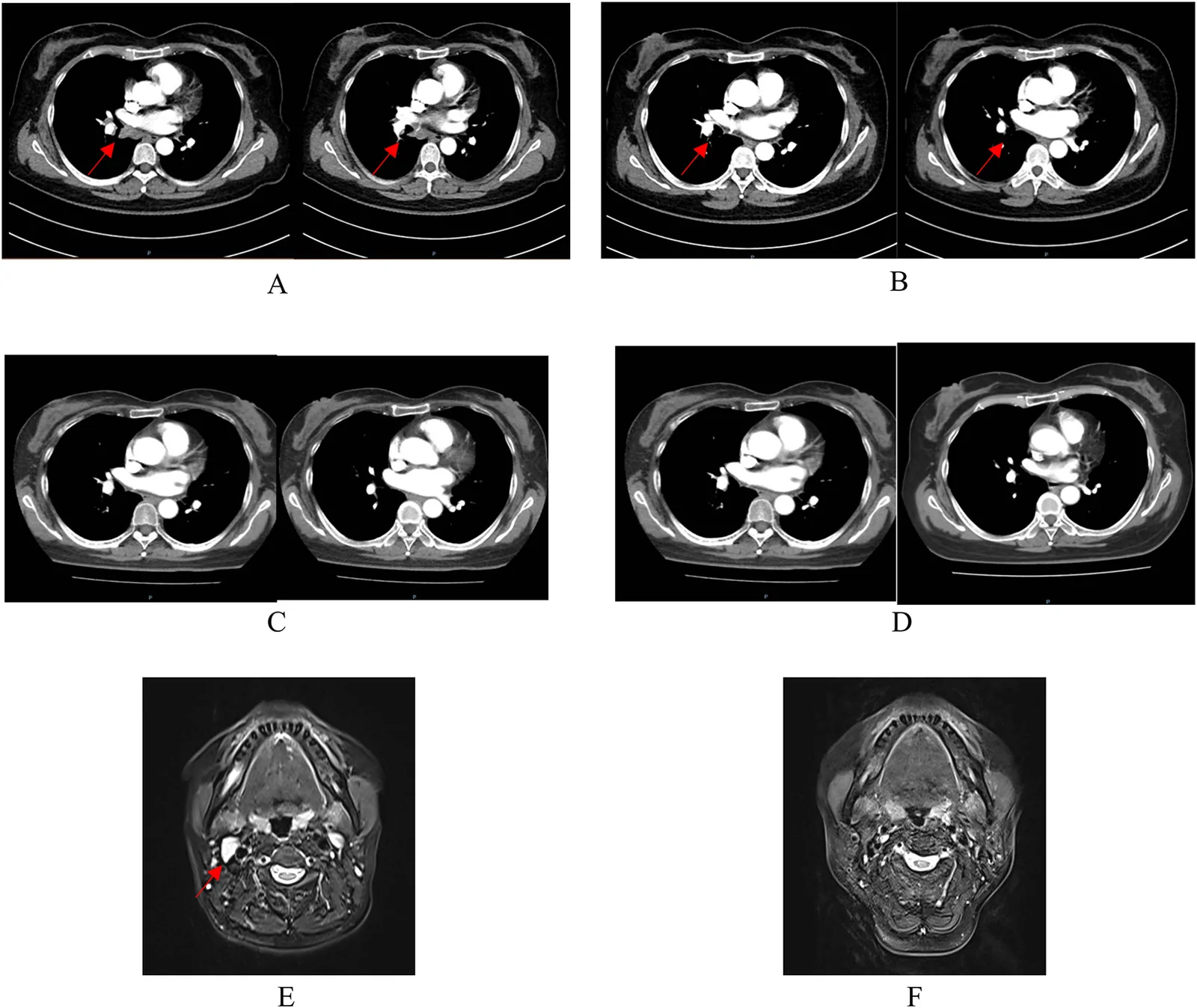
Serial imaging demonstrating thoracic and cervical tumor response. **(A)** Baseline chest CT obtained in November 2020 showing the primary right lung lesion. **(B)** Chest CT obtained after induction treatment showing marked regression of the primary lesion, consistent with a partial response. **(C)** Chest CT obtained on July 20, 2021, when a complete response was first documented according to RECIST 1.1. **(D)** Chest CT obtained on May 10, 2023, showing sustained radiological complete response. **(E)** Baseline cervical MRI obtained in November 2020 showing a representative abnormal right level II cervical lymph node. **(F)** Follow-up contrast-enhanced cervical MRI obtained on May 12, 2023, showing no pathological cervical lymphadenopathy. Arrows indicate the representative pulmonary lesion in panels A and B and the abnormal right cervical lymph node in panel E.

At initiation of antitumor therapy, her body weight was 68 kg. From November 26, 2020, to February 2, 2021, she received four 3-week cycles of serplulimab (anti-PD-1 monoclonal antibody), etoposide (topoisomerase II inhibitor), and carboplatin (platinum-based cytotoxic agent); detailed doses and schedules are summarized in **Table 1**.

**Table 1:** Principal antitumor and neurological treatments.

Period	Treatment	Dose and schedule	Outcome
Nov 26, 2020–Feb 2, 2021	Serplulimab + etoposide + carboplatin	Serplulimab 4.5 mg/kg (306 mg), day 1; etoposide 100 mg/m² (170 mg), days 1–3; carboplatin AUC 5 (560 mg), day 1; every 3 weeks for 4 cycles	PR
Feb 23–Jun 8, 2021	Serplulimab maintenance	Same serplulimab dose and 3-week schedule as above; 5 cycles	CR first documented on Jul 20, 2021
Jun 17, 2021	Methylprednisolone + IVIG	Methylprednisolone 500 mg/day ×3 days, 250 mg/day ×2 days, then 80 mg/day ×2 days; IVIG 22.5 g/day ×5 days	Rapid improvement in somnolence
After discharge	Oral methylprednisolone	40 mg/day, tapered by 4 mg weekly until discontinuation	Continued neurological improvement
Jul 19, 2021	Second IVIG course	22.5 g/day ×5 days	Neurological stabilization; slight reduction in thalamic MRI abnormalities
Jul 2021–2022	Maintenance IVIG	15 g/day ×3–5 days every 2 months	Gradual neurological improvement

At initiation of antitumor therapy, the patient’s body weight was 68 kg. At the time of neurological treatment, her body weight had decreased to 55 kg; the initial IVIG course therefore corresponded to a cumulative dose of approximately 2.0 g/kg. AUC, area under the curve; PR, partial response; CR, complete response; IVIG, intravenous immunoglobulin.

A partial response was achieved according to Response Evaluation Criteria in Solid Tumors version 1.1 (RECIST 1.1). Treatment-related adverse events included nausea, constipation, myelosuppression, alopecia, and hypothyroidism, which was controlled with levothyroxine.

Serplulimab maintenance therapy was initiated on February 23, 2021. Five maintenance cycles were administered in total, with one scheduled cycle temporarily withheld after neurological symptoms first emerged.

During the early maintenance phase, the patient developed anorexia, weight loss, and alternating constipation and diarrhea. Colonoscopy showed intestinal edema, whereas gastroduodenoscopy revealed no significant abnormality. No intestinal biopsy was obtained. At that time, the symptoms were considered to represent a functional bowel disorder rather than an ICI-related adverse event, and only symptomatic treatment was provided.

After the maintenance dose administered on April 6, 2021, she developed progressive memory impairment and depressed mood and was diagnosed with mild depression at another institution. One scheduled serplulimab cycle was withheld. Her symptoms partially improved after antidepressant treatment. Although memory impairment persisted, it continued to be attributed to depression rather than immune-mediated neurological toxicity. Because an immune-mediated neurological adverse event was not suspected at that time, the fifth serplulimab maintenance dose was administered on June 8, 2021, after discussion with and agreement from the family. One day later, she developed marked somnolence, worsening cognitive dysfunction, and gait instability.

Neurological examination revealed somnolence, slowed speech, and marked impairment of memory, calculation, and orientation. Extraocular movements were intact, pupils were equal and reactive to light, and limb muscle strength and tone were preserved. Romberg testing was recorded as positive; however, given the patient’s somnolence and cognitive impairment, the reliability of this finding was limited. It was therefore interpreted cautiously as evidence of postural instability rather than as proof of peripheral neuropathy. Sensory and coordination examinations could not be completed reliably because of impaired cooperation. No definite peripheral neuropathy was documented in the available follow-up records.

During the acute encephalitic episode, cognitive screening demonstrated substantial impairment, with a Montreal Cognitive Assessment (MoCA) score of 11/30 and a Mini-Mental State Examination (MMSE) score of 12/30. The modified Rankin Scale (mRS) score was 3.

## 3 Diagnostic assessment

Cerebrospinal fluid (CSF) examination showed a leukocyte count of 16 × 10^6^/L, comprising 97% mononuclear cells and 3% polymorphonuclear cells. CSF glucose was 3.2 mmol/L, protein was 0.54 g/L, and chloride was 115.3 mmol/L. Paired serum glucose was measured and was within the reference range. Oligoclonal band testing showed no abnormality, and CSF cytology was negative for malignant cells.

CSF microbiological investigations, including herpes simplex virus and varicella-zoster virus polymerase chain reaction testing and other available pathogen studies, were negative.

At the onset of neurological deterioration, serum sodium was 137 mmol/L. Serum potassium and calcium, liver and renal function tests, and thyroid function were within their respective reference ranges.

A 14-antibody autoimmune encephalitis panel was negative. Anti-Hu antibody was detected in CSF using a cell-based indirect immunofluorescence assay (cell-based assay, CBA) at a titer of 1:100, with testing performed at Guangzhou Kingmed Diagnostics Group Co., Ltd. The remaining antibodies in the paraneoplastic neurological antibody panel were negative. The anti-Hu result was not independently confirmed using a second assay, and no residual CSF or paired archived specimen was available for repeat or orthogonal confirmatory testing. Therefore, the antibody result was interpreted as supportive rather than independently diagnostic of anti-Hu-mediated encephalitis.

Electroencephalography performed on June 22, 2021, demonstrated diffuse background slowing with abundant irregular delta activity over the bilateral frontal and temporal regions.

Brain MRI before cancer treatment showed a left frontal ischemic lesion, while MRI in May 2021 demonstrated bilateral frontal ischemic changes (**Figures 3A, B**), providing a background of pre-existing cerebrovascular disease. MRI obtained during acute neurological deterioration on June 16, 2021, demonstrated newly developed bilateral paramedian thalamic signal abnormalities (**Figure 3C**). Given the lesion distribution and the pre-existing ischemic lesions, a vascular etiology, including artery of Percheron infarction, was specifically considered. Diffusion-weighted imaging (DWI) and apparent diffusion coefficient (ADC) mapping showed no new diffusion-restricted lesion or corresponding ADC reduction (**Figures 3D, E**). Magnetic resonance angiography (MRA) showed no major intracranial arterial occlusion or other evident culprit vascular abnormality. Although routine MRA may not directly visualize the small artery of Percheron, the combined imaging findings argued against an acute infarction.

**Figure 3:**
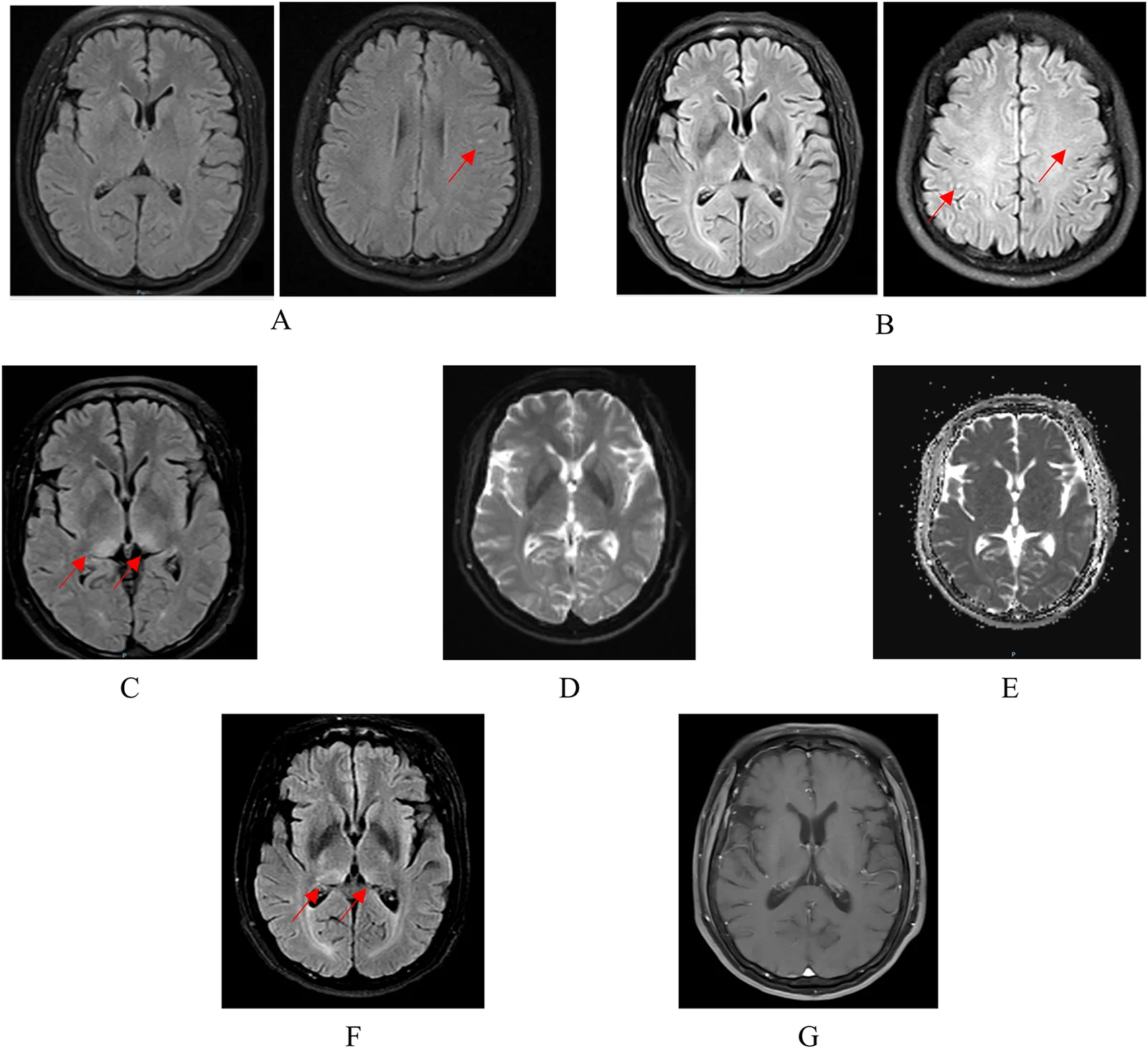
Serial brain magnetic resonance imaging findings during the neurological course. **(A)** Representative axial T2-FLAIR images obtained on November 21, 2020, showing a left frontal ischemic lesion at the frontal level and no obvious abnormality at the corresponding thalamic level. **(B)** Representative axial T2-FLAIR images obtained in May 2021, showing bilateral frontal ischemic changes at the frontal level and no obvious abnormality at the corresponding thalamic level. **(C)** Axial T2-FLAIR MRI obtained on June 16, 2021, showing newly developed bilateral paramedian thalamic hyperintensities. **(D)** Axial diffusion-weighted imaging obtained on June 16, 2021, showing no newly developed diffusion-restricted lesion. **(E)** Corresponding apparent diffusion coefficient map showing no reduction compatible with acute cerebral infarction. **(F)** Axial T2-FLAIR MRI obtained on July 20, 2021, showing a slight reduction in the bilateral paramedian thalamic abnormalities after immunomodulatory treatment. **(G)** Follow-up axial contrast-enhanced T1-weighted MRI obtained on May 12, 2023, showing no abnormal intracranial enhancement. Arrows indicate representative frontal ischemic changes and paramedian thalamic abnormalities.

Wernicke encephalopathy was also considered because of the preceding anorexia, weight loss, cognitive impairment, somnolence, and gait instability. Serum thiamine was not measured, although vitamin B supplementation was administered during treatment. Wernicke encephalopathy therefore could not be completely excluded on biochemical grounds.

No intracranial metastatic lesion was identified on MRI, and CSF cytology was negative for malignant cells. Infectious encephalitis was considered less likely in view of the negative CSF microbiological investigations. Major metabolic and electrolyte-mediated encephalopathy was also considered unlikely on the basis of the laboratory findings.

According to the 2021 PNS-Care criteria, the neurological presentation was classified as an intermediate-risk phenotype rather than a classical high-risk phenotype. If the reported CSF anti-Hu result is accepted, the recalculated PNS-Care score would be 9 (2 points for the intermediate-risk neurological phenotype, 3 points for the high-risk anti-Hu antibody, and 4 points for concordant SCLC), corresponding to the definite PNS category under the PNS-Care framework (8). However, this classification remains limited by the absence of independent confirmation of the anti-Hu result. In parallel, the clinical presentation was compatible with probable ICI-related encephalitis according to consensus definitions for neurological irAEs (9). Taken together, the syndrome was considered to reflect overlapping paraneoplastic and ICI-related neurological autoimmunity rather than either entity in isolation.

## 4 Therapeutic intervention

Given the grade 3 neurological toxicity according to the Common Terminology Criteria for Adverse Events (CTCAE) version 5.0, multidisciplinary evaluation led to the permanent discontinuation of serplulimab. High-dose intravenous methylprednisolone and IVIG were initiated, with the initial IVIG course corresponding to a cumulative dose of approximately 2.0 g/kg over 5 days. Somnolence improved substantially within 3 days, followed by partial improvement in mental status and gait instability.

Although somnolence improved rapidly after corticosteroids and the initial IVIG course, substantial cognitive impairment and gait instability persisted. The multidisciplinary team therefore administered a second course of IVIG on July 19, 2021, as additional immunomodulatory treatment before considering escalation to second-line immunosuppression. Given the subsequent neurological stabilization, maintenance IVIG was continued rather than escalating to rituximab or another second-line agent.

At our institution, severe ICI-related neurological toxicities are managed through an established multidisciplinary team (MDT) approach involving relevant specialties, with treatment escalation individualized according to neurological severity, diagnostic considerations, initial treatment response, residual neurological deficits, and subsequent clinical stability rather than according to a rigid stepwise algorithm.

## 5 Follow-up and outcomes

Tumor response was assessed according to RECIST 1.1 and incorporated both the thoracic lesion and cervical nodal disease. Ultrasonography of the superficial lymph nodes on July 19, 2021, showed no abnormal cervical lymphadenopathy. A complete response (CR) was first documented on July 20, 2021. Radiological CR remained confirmed at our institution through May 10, 2023. Contrast-enhanced cervical MRI obtained on May 12, 2023, also demonstrated no pathological cervical lymphadenopathy (**Figure 2**).

After May 2023, imaging follow-up was not performed at our institution. The patient died in January 2025 from postoperative complications following surgery for intestinal obstruction. There was no clinical evidence that tumor progression was the cause of death. Accordingly, the approximately 43-month interval between the final serplulimab dose and death is described as a period without documented clinical progression, rather than as 43 months of radiographically confirmed CR.

Neurological recovery was substantial but incomplete. At the most recent formal neurological assessment on May 12, 2023, the MoCA score improved from 11/30 to 24/30 and the MMSE score from 12/30 to 22/30; however, residual cognitive impairment persisted despite marked improvement from the acute episode. The mRS score improved from 3 to 2. Somnolence had markedly improved, and the patient was largely independent in activities of daily living. Follow-up brain MRI showed no abnormal intracranial enhancement (**Figure 3G**).

In August 2024, the patient presented with a 3-day history of obstipation and inability to pass flatus. Intestinal obstruction was clinically suspected, and segmental colectomy was subsequently performed. Examination of the resected 30-cm colonic segment showed marked dilatation of the proximal bowel, bowel-wall thickening, and substantial intraluminal fluid retention, whereas the distal bowel caliber was preserved. No tumor, fecalith, stenotic ring, adhesion, or other mechanical cause of obstruction was identified, supporting the diagnosis of intestinal pseudo-obstruction. Notably, microscopic examination demonstrated diffuse lymphocytic infiltration surrounding the myenteric plexus, suggesting an inflammatory enteric neuropathic process. At the time of the 2024 gastrointestinal episode, the patient had received no cytotoxic chemotherapy for more than 3 years and was not taking opioids, anticholinergic agents, or other medications known to impair gastrointestinal motility. Thyroid function was within the reference range, and she had no history of abdominal surgery before 2024. Anti-Hu antibody testing was not repeated during this episode. The pathological findings supported an inflammatory process involving the region of the myenteric plexus; however, the etiology remained uncertain, and a direct causal relationship with anti-Hu autoimmunity could not be established.

## 6 Discussion

This case highlights the diagnostic complexity of a rapidly progressive neurological syndrome with CSF anti-Hu positivity during PD-1 blockade in SCLC. It also documents a later episode of intestinal pseudo-obstruction with myenteric inflammation.

The term post-ICI PNS is particularly useful in this clinical setting. Anti-Hu-associated PNS is strongly linked to SCLC, whereas PD-1 blockade may amplify latent paraneoplastic immunity by releasing inhibitory constraints on autoreactive T cells (3–5, 10). Consequently, neurological syndromes emerging after ICI exposure cannot always be reliably classified as either spontaneous PNS or conventional n-irAEs. In the present patient, the underlying SCLC, CSF anti-Hu positivity, inflammatory neurological syndrome, and temporal relationship with serplulimab exposure support this overlapping interpretation.

Hu antigens are intracellular neuronal proteins, and anti-Hu antibodies themselves are not generally considered the principal mediators of neuronal injury. Cytotoxic T-cell responses directed against shared tumor-neuronal antigens are thought to have a central pathogenic role (3, 7, 10). Furthermore, anti-Hu antibodies can occasionally be detected in patients with SCLC without overt neurological manifestations (7). Anti-Hu positivity alone should therefore not be regarded as evidence of active neuronal injury.

Pretreatment serum and CSF samples were unavailable; therefore, whether serplulimab induced anti-Hu autoimmunity *de novo* or amplified pre-existing clinically silent paraneoplastic immunity cannot be determined.

Artery of Percheron infarction was specifically considered because of the lesion distribution and the patient’s pre-existing ischemic lesions. DWI/ADC showed no acute diffusion-restricted lesion, and MRA showed no major culprit vascular abnormality, although routine MRA may not directly visualize the small artery of Percheron. Wernicke encephalopathy was also clinically plausible because of the preceding anorexia, weight loss, cognitive impairment, somnolence, and gait instability. Because thiamine concentrations were not measured, Wernicke encephalopathy could not be definitively excluded biochemically. Nevertheless, the inflammatory CSF profile, CSF anti-Hu positivity, EEG abnormalities, negative infectious work-up, absence of an acute ischemic lesion, and subsequent clinical course collectively favored an immune-mediated neurological syndrome.

The gastrointestinal findings require cautious interpretation. The alternating constipation and diarrhea in 2021 occurred relatively close to systemic platinum-etoposide chemotherapy and were not histologically characterized. Colonoscopy showed intestinal edema, but no biopsy was obtained; therefore, treatment-related gastrointestinal dysfunction, a functional disorder, and early autonomic or enteric involvement could not be distinguished.

The 2024 episode was clinically different. Intestinal pseudo-obstruction occurred more than 3 years after cytotoxic chemotherapy, when the patient was not receiving chemotherapy or medications known to impair gastrointestinal motility. No mechanical obstructive lesion was identified, and histopathology demonstrated lymphocytic inflammation surrounding the myenteric plexus, supporting an inflammatory enteric neuropathic process. Although anti-Hu-associated enteric neuropathy and ICI-associated gastrointestinal pseudo-obstruction have been reported (6), these precedents do not establish the etiology in this patient. Nevertheless, anti-Hu testing was not repeated, neuronal-specific immunohistochemistry was not performed, and formal autonomic testing was unavailable. Prior chemotherapy therefore remains an alternative contributing factor rather than an excluded cause. The 2024 findings should consequently be regarded as suggestive of possible enteric nervous system involvement, not as proof of anti-Hu-mediated enteric neuropathy.

The prolonged tumor control after ICI discontinuation remains clinically noteworthy. However, it is presented here as a parallel oncological outcome rather than as evidence of a mechanistic association between neurological toxicity and antitumor efficacy. No immune profiling was performed that could establish a biological link between these two observations.

Permanent ICI discontinuation and prompt high-dose corticosteroids plus IVIG were followed by rapid improvement in somnolence and gradual neurological recovery. However, treatment response does not distinguish PNS from n-irAE because both are immune-mediated. Recovery remained incomplete despite improvement in MoCA, MMSE, and mRS scores. In retrospect, the persistence of cognitive symptoms before the fifth serplulimab maintenance dose represented a missed opportunity for earlier recognition of possible neurological immune toxicity. At the time, however, the symptoms had been attributed to depression and had partially improved with antidepressant treatment.

In patients with SCLC receiving ICIs who develop cognitive, behavioral, gait, or unexplained gastrointestinal abnormalities, evaluation should include brain MRI with diffusion sequences, CSF examination, appropriate microbiological testing, paraneoplastic and autoimmune antibody testing, and EEG when clinically indicated (5, 8, 9). Anti-Hu positivity should be interpreted in conjunction with ICI exposure, neurological phenotype, imaging, CSF findings, and competing diagnoses rather than as definitive evidence of classical PNS.

Several limitations should be acknowledged. First, the CSF anti-Hu antibody was detected by a single CBA at one external laboratory and was not independently confirmed by an orthogonal assay; no residual specimen was available for repeat testing. Second, pretreatment anti-Hu status and serial antibody titers were unavailable. Third, the 2021 gastrointestinal symptoms were not biopsied, and anti-Hu testing, neuronal-specific immunohistochemistry, and formal autonomic testing were not performed during the 2024 pseudo-obstruction episode. Prior chemotherapy therefore remains an important alternative contributor to gastrointestinal dysfunction. Fourth, serum thiamine was not measured during the acute neurological episode. Fifth, although MRA showed no major intracranial arterial occlusion or evident culprit vascular abnormality, routine MRA may not directly visualize the small artery of Percheron. Finally, no T-cell phenotyping, T-cell receptor sequencing, serial cytokine profiling, or neuronal injury biomarkers were performed.

Despite these limitations, this case illustrates the diagnostic complexity of neurological syndromes emerging during ICI therapy in SCLC, particularly when paraneoplastic autoimmunity and ICI-related neurotoxicity may overlap. The later gastrointestinal episode raises, but does not establish, the possibility of enteric nervous system involvement.

## 7 Patient perspective

Written informed consent for publication of this case report and the accompanying clinical images was obtained from the patient’s family/next of kin after the patient’s death.

According to the family, the patient and her relatives considered that serplulimab-based therapy had provided substantial tumor control. At the same time, they emphasized the importance of awareness of potentially serious immune-related neurological complications in patients with neuroendocrine malignancies receiving immunotherapy. They hoped that publication of this case would increase clinical awareness and facilitate earlier recognition and management of neurological and gastrointestinal manifestations.

## 8 Conclusion

A rapidly progressive neurological syndrome with CSF anti-Hu positivity developed during serplulimab treatment for ES-SCLC and was most appropriately interpreted as an overlap between paraneoplastic neurological autoimmunity and ICI-related neurotoxicity. The later intestinal pseudo-obstruction with lymphocytic inflammation surrounding the myenteric plexus raised the possibility of enteric nervous system involvement, although a specific anti-Hu-mediated mechanism could not be established. Neurological recovery was substantial but incomplete. The prolonged tumor control after ICI discontinuation was a separate noteworthy oncological outcome.

## Data Availability

The datasets presented in this article are not readily available because this is a single-patient case report, and no separate research dataset was generated. All relevant anonymized clinical data are included in the article. Further inquiries can be directed to the corresponding author.
